# Assessment of heterocyclic aromatic amines contents in flamed and braised chicken in Burkina Faso

**DOI:** 10.1371/journal.pone.0278712

**Published:** 2022-12-30

**Authors:** Bazoin Sylvain Raoul Bazié, Adjima Bougma, Aminata Séré, Judicael Thomas Ouilly, Hassane Sangaré, Elie Kabré, Aly Savadogo, Djidjoho Joseph Hounhouigan, Marie-Louise Scippo, Imaël Henri Nestor Bassole

**Affiliations:** 1 Laboratoire de Biologie Moléculaire d’Épidémiologie et de Surveillance des agents Transmissibles par les Aliments (LABESTA), Unité de Formation et de la Recherche en Sciences de la Vie et de la Terre, École Doctorale Sciences et Technologies, Université Joseph Ki-Zerbo, Ouagadougou, Burkina Faso; 2 Laboratoire National de Santé Publique, Ouagadougou, Burkina Faso; 3 Laboratoire de Biochimie et d’Immunologie Appliquée, Unité de Formation et de la Recherche en Sciences de la Vie et de la Terre, Université Joseph Ki-Zerbo, Ouagadougou, Burkina Faso; 4 Laboratoire de Sciences des Aliments, Faculté des Sciences Agronomiques, Université d’Abomey, Cotonou, Benin; 5 Département des Sciences des Denrées alimentaires, Centre de recherche FARAH–Secteur Santé Publique Vétérinaire, Liège, Belgique; Ataturk Universitesi, TURKEY

## Abstract

The nutritional status of meat is tarnished by its association with the induced cooking contaminants. The aim of this study was to assess the heterocyclic aromatic amines profile and contents in processed chicken in Burkina Faso. Eight polar and apolar heterocyclic aromatic amines (HAAs) including 2-mino-3-methylimidazo[4,5-f]quinolone (IQ), 3-amino-1,4-dimethyl-5H-pyrido[4, 3-b]indole (Trp-P1), 3-amino-1-methyl-5H-pyrido[4,3-b]indole (Trp-P2), 2-mino-9H-pyrido-[2,3-b]indole (AαC), 2-amino-1-methyl-6-phenylimidazo[4, 5– ]pyridine (PhIP), 2-amino-3-methyl-9H-pyrido[2,3-b] indole (MeAαC), 2-amino-3,4,8-rimethylimidazo[4,5-f]quinoxaline (4,8-DiMeIQx) and 2-amino-3,8-imethylimidazo[4,5–]quinoxaline (MeIQx) were screened by high performance liquid chromatography from 29 samples of flamed chicken and 66 samples of braised chicken collected in Ouagadougou city. Apolar HAAs and polar HAAs were respectively 12 and 3 times more abundant in flamed chickens (32.66±10 and 3.48±10.39 ng/g, respectively) than in braised chickens (2.70±9.67 and 0.92 ng/g, respectively). The maximum levels of AαC were in the same proportions in flamed (12.01 ng/g) and braised chickens (14.13 ng/g). Flamed chicken had the highest Trp-P1 content (530.31 ng/g). The 4,8-DiMeIQx was not detected in braised chicken. The AαCs were more abundant in flamed than in braised chicken. The profile and the contents of the HAAs in processed chicken are related to cooking methods. Because of the high variability observed on the obtained concentrations, investigations on the contents of precursors in raw chicken, the effect of marinating ingredients on the formation of HAAs are needed.

## Introduction

Meat is an important source of protein, essential amino acids, essential minerals and vitamins. However, the healthy and nutritious image of meat is often tarnished by its saturated fatty acid composition, cholesterol content, and especially the presence of micropollutants and toxic substances induced by processing and cooking [1]. One of the major contaminants induced by meat processing issues are heterocyclic aromatic amines [1]. Heterocyclic aromatic amines (HAAs) are compounds that form naturally in muscle tissue when exposed to heat during the cooking process [2, 3]. They are classified into two groups, depending on their formation process. The HAAs formed at temperatures between 100°C and 300°C are referred to as thermal, IQ-type HAAs (imidazoquinoline), or aminoimidazoazarenes, and others formed at higher temperatures, above 300°C, are referred to as pyrolytic, or non-QI-type HAAs [4].

Thermal HAAs are generated by the reaction of free amino acids, creatine and hexoses. In the case of non-IQ type HAAs, the formation occurs by the pyrolytic reaction between amino acids and proteins.

The formation of HAAs depends on several parameters such as the concentration of precursors like amino acids, creatine and sugar [5].

Studies have shown that the abundance of formed HAAs is positively correlated with the concentration of precursors such as glucose, creatine and free amino acids [5]. The type of precursors contained in the meat determines the amount and type of HAAs formed during cooking. Indeed, in meat rich in phenylalanine, tyrosine and isoleucine such as chicken, the formation of 2-amino-1-methyl-6-phenylimidazo[4, 5– ]pyridine (PhIP) is more important [6]. This is due to the fact that these three amino acids are precursors for PhIP formation [7].

Both temperature and cooking time affect the yields of HAAs formation [8]. However, the influence of cooking temperature appears to be more important than that of cooking time. For example, cooking at temperatures below 300°C strongly decreases the formation yields of carbolines formed exclusively by thermal mechanisms at temperatures above 300°C [8]. In addition, kinetic studies performed on the formation of HAAs showed that the amounts of HAAs formed quickly reached a maximum, and more rapidly at higher temperatures [9].

Since the first discovery of Sugimura et al. [3], more than thirty (30) different HAAs have been identified in protein-rich cooked foods [10].

The International Agency for Research on Cancer (IARC) has classified one HAA, IQ, in Group 2A (probably carcinogenic to humans) and nine others, MeIQ, MeIQx, PhIP, AαC, MeAαC, Trp-P-1, Trp-P-2, Glu-P-1, and Glu-P-2, as Group 2B (possibly carcinogenic to humans) [11–13]. Studies have demonstrated that regular intake of grilled meat with HAAs may enhance the risk of cancer in different organs of humans [14]. It has been recommended that the exposure of these components to humans should be lessened [15]. Numerous studies on the content of HAAS in poultry meat, mainly chicken, have been published. Salmon et al (2005) [2] reported the presence of HAAs in chicken meat from domestic preparation of Chinese cuisine in Singapore. Liao et al (2010) [16] and Oz et al (2010) [17] have shown the effects of cooking methods on the formation of HAAs in chicken meat. Indeed, the cooking method can influence the formation of HAAs, because the heat transfer to the meat, the temperature gradient in the meat varies according to the cooking methods [18]. Cooking with or without the skin in the case of poultry or fish is also a factor modifying the temperature gradient. Cooking meat in slices results in an increase in the heat exchange surface and thus in the quantities of HAAs formed [19]. In addition, since the precursors of HAAs are hydrophilic, they can be carried away by water migration phenomena within the meat during cooking [20].

In Burkina Faso, the consumption of chicken is an important part of meat consumption habits. Barro et al (2002) [21] Kagambega et al (2012) [22] and Somda et al (2018) [23] reported the presence of pathogens in processed meats sold on the street in Burkina Faso. It has been reported a chemical risk assessment of metallic trace elements and polycyclic aromatic hydrocarbons [24, 25] associated with the consumption of flamed and braised chicken in Burkina Faso. However, very little information is available on the presence of HAAs in processed chicken in Burkina Faso. The objective of this study is to assess the profile and the contents of heterocyclic aromatic amines in processed chicken in Burkina Faso.

## Materials and methods

### Sampling

**Fig 1:**
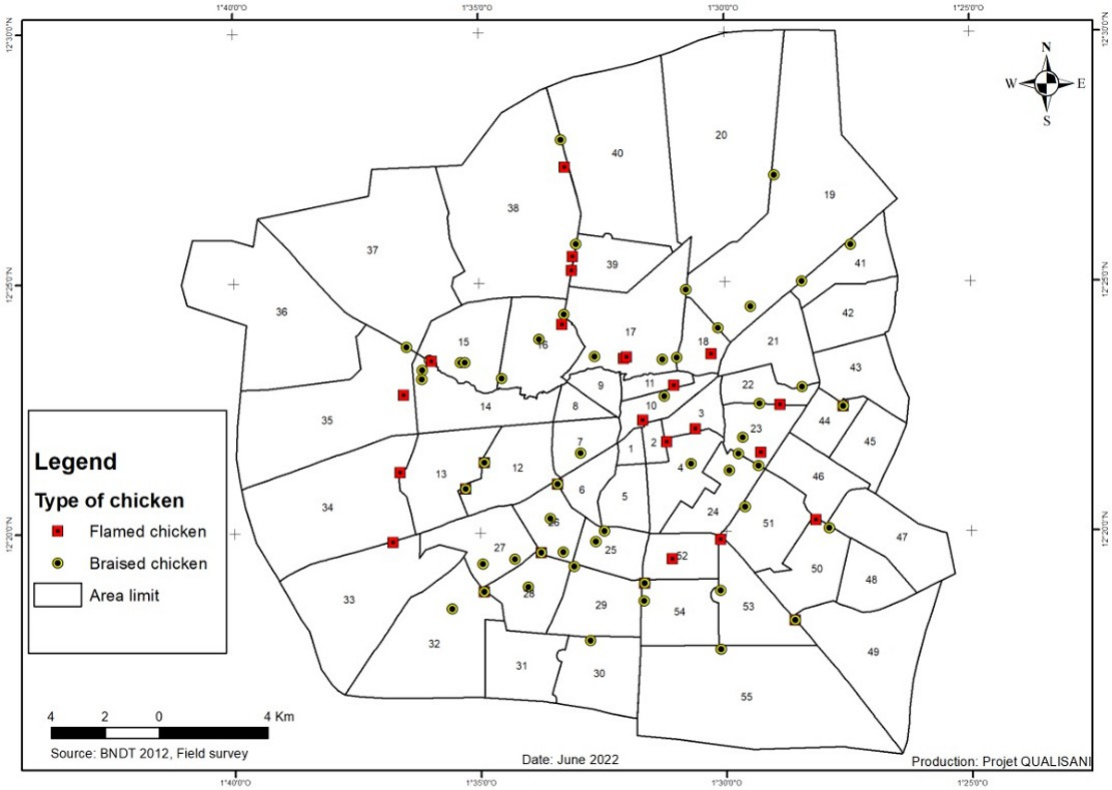
Sampling points.

The study was conducted in the city of Ouagadougou where the production and consumption are a part of the food habits. A total of 95 samples including 29 flamed chicken and 66 braised chicken meats were collected in different processing and sale points Ouagadougou city as showed in the Fig 1. The samples collection was consisting to cut the chicken into pieces by the processors. These pieces were collected and packaged in a craft paper wrapper, according to the usual conditions of purchase by the consumers. The collected samples are placed in sterile stomacher bags, labelled and stored in a cooler to maintain the temperature at about 4°C and transported to the laboratory prior for analysis.

Processing parameters such as fuel type, core temperature (thermometer KJJ004), distance from heat source, mass loss (balance Gibertini Electtronica) and cooking time were recorded for both flamed and braised processing technics.

### Reagents and standards

Solvents (dichloromethane, methanol and acetonitrile) of HPLC grade (Sigma Adrich, USA) were used for extraction and for the mobile phase. Chemical reagents, including sodium hydroxide and ammonium acetate, were purchased from Panreac (Spain). Extraction cartridges (Extrelut-20) and refill diatomaceous earth, Bond Elut PRS (500 mg) and C18 (100 mg) cartridges were provided by Starlab Scientific (China).

HAAs standards: 2-mino-3-methylimidazo[4,5-f]quinolone (IQ), 3-amino-1,4-dimethyl-5H-pyrido[4,3-b]indole (Trp-P1), 3-amino-1-methyl-5H-pyrido[4,3-b]indole (Trp-P2), 2-mino-9H-pyrido-[2,3-b]indole (AαC), 2-amino-1-methyl-6-phenylimidazo[4, 5– ]pyridine (PhIP), 2-amino-3-methyl-9H-pyrido[2,3-b] indole (MeAαC), 2-amino-3,4,8-rimethylimidazo[4,5-f]quinoxaline (4,8-DiMeIQx) 2-amino-3,8-imethylimidazo[4,5– ]quinoxaline (MeIQx) were obtained with ChemCruz (USA).

### Apparatus

A Philips stainless steel mixer (Mixer Grinder HL7810/00, India) was used to grind and homogenize the samples. A Memmert oven (Büchenbach, Germany) was used to measure the moisture content. Purification was done on a Vac-Elut Varian system (Australia). An Ultimate 3000 UHPLC system (Thermo Scientific, USA) was used for the analysis of HAAs.

### Analysis of the HAAs

#### Extraction

The extraction of heterocyclic aromatic amines was done according to the method described by Santos et al. [26].

One (1) gram of sample was weighed in a test tube and 12 ml of NaOH was added. The mixture was ultrasonicated (VWR, USC-TH Radnor, Pennsylvania, United States) for 15 minutes and stirred (VWR, HMS-6, Radnor, Pennsylvania, United States) for 60 minutes. The resulting alkaline solution was mixed with the diatomaceous earth. This mixture was reconditioned in an Extrelut cartridge where the extraction took place with 75 ml of dichloromethane.

#### Purification

The obtained extract was passed on a propyl sulfonic acid (PRS) cartridge. The PRS cartridge was then washed successively with 15 ml of methanol/water (40/60) and 2 ml of water. The HAAs retained in the PRS cartridge were eluted with 20 ml of 0.5 M ammonium acetate, pH 8.5. This eluate was run on a C_18_ cartridge. After, a wash phase of the cartridge with 5 ml of water, the HAAs were eluted with 1 ml of methanol/ammonia (9/1). The eluate was then evaporated to dryness with a nitrogen flow and the residue was recovered in 100 μl of methanol and put in vial for HPLC injection.

### Chromatographic analysis

A Thermo Scientific Ultimate 3000 liquid chromatography equipped with a DAD detector was used for identification. The quantification of HAAs was performed under the following chromatographic conditions: Analytical column Zorbax SB C18 (5m, 5mm x 250mm) Agilent technologies was used with a mobile phase constituted by Methanol/Acetonitrile/Water/Acetic acid (8/14/76/2 v/v/v), pH adjusted to 5.0 with ammonium hydroxide (Solvent A) and Acetonitrile (Solvent B). The elution gradient was set as follows: 6%B 0 min, 9%B 0-1min, 15%B 1–3 min, 70%B 3–4.5 min 70%B 4.5–5.5 min 6%B 5.5 min. The detector wavelength was 262 nm.

The retention times of the standards were used for the identification of HAAs. Quantification was done according to the external standard method.

### Quality control

A procedural blank and a spiked blank matrix were introduced in each injection series for quality control to check the efficiency of the different steps of the analysis by calculating the recovery rates.

The determination of the limits of detection (LOD) was based on the 3:1 ratio of signal to noise of the lowest spiked matrix. The parameters of method performance are presented in the Table 1.

**Table 1:** The parameters of method performance.

HAAs	LOD (ng/g)	Recovery (%)	Coefficient (R^2^)
**AαC**	0.6	70	99.90
**MeAαC**	0.1	40	99.25
**MeIQx**	0.2	60	99.89
**IQ**	0.2	50	99.84
**PhIP**	0.2	60	99.97
**4,8-DiMeIQx**	0.6	60	99.95
**TrP-P1**	0.6	60	99.88
**Trp-P2**	0.6	70	99.96

Analyses were performed in duplicate and recoveries based on spiked samples were ranged from 40 to 70%. These recoveries were similar to those obtained by Melo et al. [27].

### Statistical analysis

Statistical analyses were performed using SPSS version 23 software (IBM SPSS, Armonk, NY, USA). Data were tested for normal distribution and homogeneity of variances using the Shapiro-Wilk and Levene tests, respectively.

## Results and discussion

### Processing parameters of flamed chicken and braised chicken

**Fig 2:**
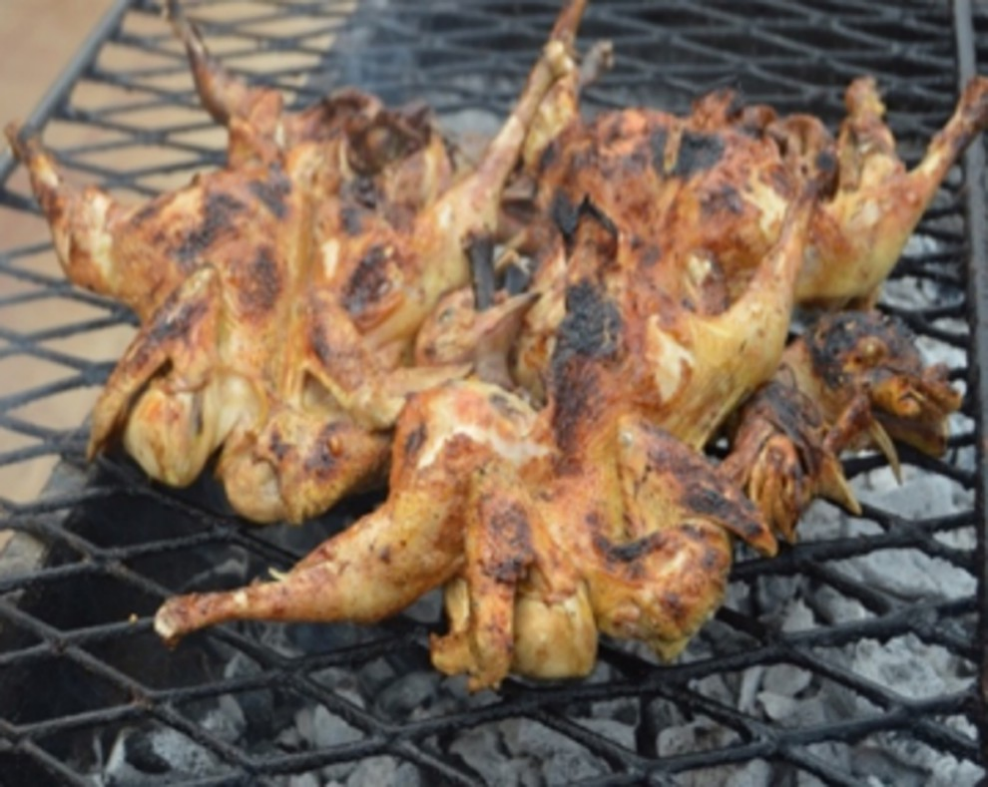
Braised chicken.

Braised chickens (Fig 2) are cooked over glowing charcoal. The wood from which the charcoal was made was not always known, but *vitellaria paradoxa* was cited as the dominant and preferred wood by processors. The plucked chicken ready for braising was seasoned (oil, salt, and garlic, unidentified spices) and placed on an iron grill about 5 cm from the glowing charcoal. Cooking time was estimated at 45 ±10 minutes for a core temperature of 93±10°C.

**Fig 3:**
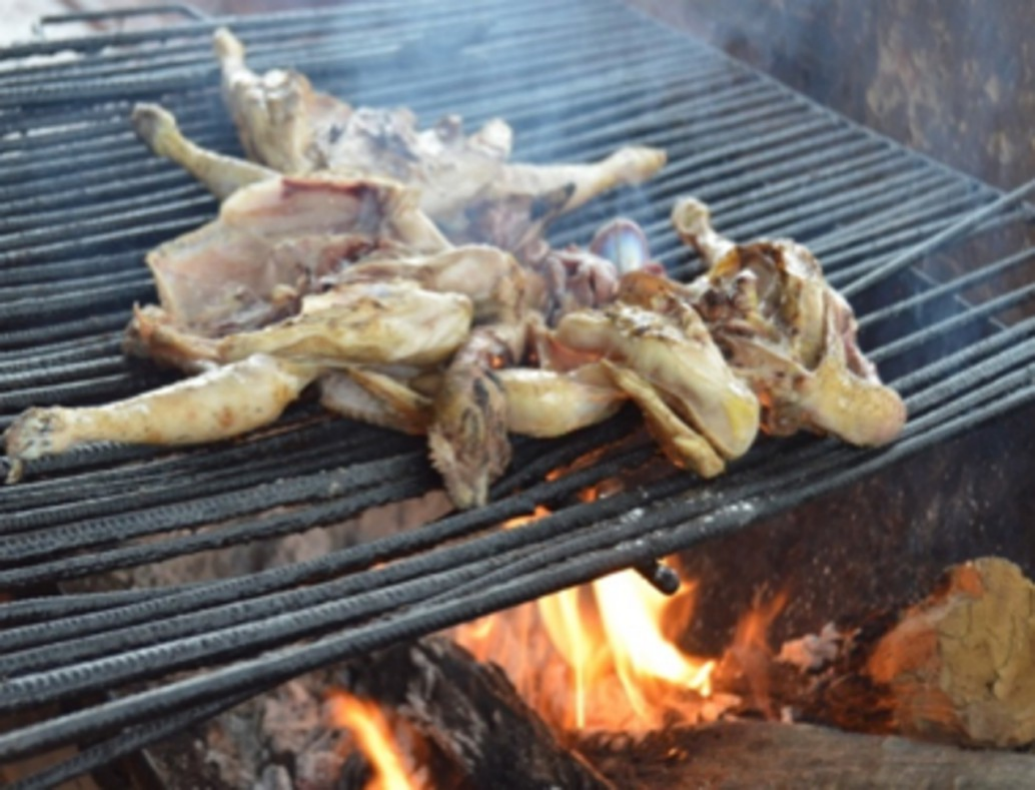
Flamed chicken.

For the flamed chicken (Fig 3) wood was the fuel used, and came mainly from *Parkia biglobosa* and *Vitellaria paradoxa* tree according to the processors. The chickens, seasoned with oil, salt, garlic and food broth, were placed on an iron grill and cooked with the flames of the wood fire.

The distance between the flame and the chickens was estimated at 3 cm and the cooking time at 25±5 minutes for a core temperature of 90±9°C (Table 2).

**Table 2:** Processing parameters of flamed and braised chicken [2,5].

	Fuel	Core temperature (°C)	Distance to heat source (cm)	Mass loss (%)	Cooking time (min)
**Flamed chicken**	Wood	90±9	3±1	29.40±5.63	25±5
**Braised chicken**	Charcoal	93±10	5±2	37.71±5.06	45±10

Cooking temperatures of braised and flamed chicken were above 500°C and core temperatures of the products ranged from 93±10°C (braised chickens) to 90±9°C (flamed chickens) with respective cooking times of 25 minutes and 45 minutes. The formation of HAAs is dependent on heating time and heating temperature depending on the cooking method used [28].

The heating temperature has the most significant effect on the formation of HAAs. According to Ahn and Grün (2005) [29], few or no HAAs were detected at a temperature of 160°C for a heating time of 15 minutes. The influence of cooking temperature appears to be more important than that of cooking time [8]. In addition, kinetic studies on the formation of HAAs showed that the amounts of HAAs formed reached a maximum rapidly, and the higher the temperature, the faster they were formed. It has also been shown that at certain temperatures, HAAs can be degraded [8]. However, it should be noted that although a lot of research has been devoted to study the influence of different cooking parameters (especially temperature and time) on the quantities of HAAs formed, it is often difficult to compare the values of the results obtained because the methods of measuring these cooking parameters are not harmonized [4].

The obtained results did not follow a normal distribution (Shapiro-Wilk test, p<0.05). The contents of polar and apolar heterocyclic aromatic amines are presented in Tables 3 and 4 respectively.

**Table 3:** Concentration of polar heterocyclic aromatic amines.

		MeIQx ng/g	IQ ng/g	4,8-DiMeIQx ng/g	PhIP ng/g	Σ polar HAAs ng/g
**Flamed chicken**	**Med**	0.89±0.39^a^	0.52±0.18^a^	0.08±0.01^b^	2.05±1.10^a^	3,48±10,39
	**Min**	nd	nd	nd	nd	nd
	**Max**	19.71	4.85	0.28	57.37	81,92
	**Freq(%)**	28.57	50.00	7.14	3.57	72,41
**Braised chicken**	**Med**	0.17±0.26^b^	0.30±0.12^a^	nd	0.45±0.74^b^	0,92±1,64
	**Min**	nd	nd	nd	nd	nd
	**Max**	4.47	7.08	nd	3.21	11,55
	**Freq(%)**	20.63	33.33	0	39.68	59,09

Med, Mediane value; Min, Minimum value; Max, Maximum value; Freq, Frequency; nd, not detected; Median followed by same superscript letters a, b indicates no significant difference (p < 0.05) Mann-Whitney U test in the same line

**Table 4:** Concentration of apolar heterocyclic aromatic amines.

		Trp-P2 ng/g	Trp-P1 ng/g	AαC ng/g	MeAαC ng/g	Σ apolar HAAs ng/g
**Flamed chicken**	**Med**	0.27±0.11^a^	31.62±10.84^a^	0.70±0.4^a^	0.07±0.12^b^	32,66±10
	**Min**	nd	nd	nd	nd	nd
	**Max**	4.67	530.31	12.01	2.00	530.31
	**Freq (%)**	7.14	25.00	14.28	3.57	37,93
**Braised chicken**	**Med**	0.05±0.07^b^	1.70±7.23^b^	0.57±0.26^a^	0.38±0.08^a^	2,70±9,67
	**Min**	nd	nd	nd	nd	nd
	**Max**	1.36	44.73	14.13	2.94	61,24
	**Freq (%)**	11.11	11.11	15.87	25.39	53,03

Med,Mediane value;Min, Minimum value; Max, Maximum value; Freq, Frequency; nd, not detected; Median followed by same superscript letters a, b indicates no significant difference (p < 0.05) Mann-Whitney U test in the same line

### Concentration of polar HAAs in flamed and braised chicken

The contents of the sum of polar HAAs ranged from not detected to 81.92 ng/g, and from not detected to 11.55 ng/g in flamed and braised chickens, respectively.

The median value of the sum of polar HAAs was three times higher in flamed chicken (3.48±10.39 ng/g) than in braised chicken (0.92 ng/g). The 2-amino-3-methylimidazo[4,5-f]quinolone (IQ) and 2-amino-1-methyl-6-phenylimidazo[4,5–]pyridine (PhIP) were the most detected polar HAAs in flamed chickens (50%) and braised chickens (39.68%).

A 65% occurrence frequency of 2-amino-3-methylimidazo[4,5-f]quinolone (IQ) was reported by Oz et al (2010) [17] in broiled chicken samples at a mean concentration of 8 ng/g in Turkey. Busquets et al. [30] reported a frequency of 0% IQ in grilled chicken samples cooked at 175–200°C for 13 minutes in Spain. The core temperatures of the products were 93±10°C and 90±9°C for braised and flamed chickens, respectively. These temperatures were lower than those reported in the study of Busquets et al. [30]. Indeed, it has been shown that above a certain temperature, HAAs can be degraded [8].

The 2-amino-1-methyl-6-phenylimidazo[4, 5–]pyridine (PhIP) was the most abundant polar HAAs. The highest concentration was recorded in the flamed chicken samples (57, 37 ng/g). Liao et al (2010) [16] showed that PhIP was formed more readily in chicken than in beef, pork or fish during cooking and is generally found at higher levels than other HAAs in chicken [31–34]. Higher concentrations of PhIP than those found in the present study have been reported by Sinha et al (480 ng/g) [33], Salmon et al (330ng/g) [35], Knize et al (270 ng/g) [36] in barbecued chickens. However, some authors have reported the absence of PhIP in grilled chickens [33, 34, 37]. This variability in PhIP content could be related to the different processing parameters on the one hand, but especially to the content of certain amino acids such as phenylalanine, tyrosine and isoleucine in raw chickens on the other hand. Indeed, the formation of PhIP in meat is proportional to the abundance of these amino acids [6]. This is due to the fact that these three amino acids are precursors for PhIP formation [7].

Maximum levels of 2-amino-3,8-imethylimidazo[4,5–]quinoxaline (MeIQx) ranged from 4.47 ng/g (braised chickens) to 19.71 ng/g (flamed chickens). A high MeIQx content of more than 100 ng/g in chicken muscle flame-grilled for 6 minutes was reported by Holder et al. (1977) [38].

2-Amino-3,4,8-rimethylimidazo[4,5-f]quinoxaline (4,8-DiMeIQx) was present in 7.4% of the flamed chicken samples at a median concentration of 0.08 ng/g. According to the results of some studies, MeIQx and 4,8-DiMeIQx were not detected in muscle samples of fried and charcoal-grilled chickens [39], similar results were obtained for braised chickens from Burkina Faso.

### Concentration of apolar HAAs in flamed and braised chickens

The detection frequencies of apolar heterocyclic aromatic amines in the flamed and braised chicken samples from 3.57% to 25.39%. Apolar HAAs were 12 times more abundant in flamed chickens (32.66±10 ng/g) than in braised chickens (2.70±9.67 ng/g).

Trp-P1 and Trp-P2 were detected in the same frequencies (11.11%) in braised chickens. Trp-P1 was 3 times more frequent than Trp-P2 in the flamed chicken samples.

Significant differences were observed for the levels of MeIQx, 4,8-DiMeIQx, PhIP, Trp-P1, TrP-P2 and MeAαC in flamed and braised chickens. The flamed chickens had the highest Trp-P1 content (530.31 ng/g). The median concentration (31.62±10.84 ng/g) was more than 18 times higher than that found in braised chickens.

The maximum levels of AαC in flamed (12.01 ng/g) and braised (14.13 ng/g) chickens were similar. However, higher levels of AαC were reported in broiled chicken (180.4 ng/g [40]). Furthermore, the concentration of 170 ng/g in grilled chicken at cooking temperatures above 350°C [41] and content of more than 100 ng/g in slices of chicken prepared over an open flame have been reported [42].

For both polar and apolar groups, flamed chicken showed the higher contents than braised one, this can be explained by the fact that in case of flaming, the chicken is in direct contact with the flame, resulting a higher cooking temperature. Indeed, HAAs concentrations are higher in well-done meat and meat products cooked at high temperatures [43].

The cooking method can influence the formation of HAAs, as the heat transfer to the meat and the temperature gradient in the meat vary with different cooking methods [18]. Similarly, regular turning of the meat during cooking can decrease the formation of HAAs by changing the temperature gradient in the meat [44].

The use of spices (rosemary, thyme, sage, garlic, brine [45, 46], red yeast rice (Monscus red) and marjoram as well as pine bark and grape seed extracts have also been shown to disrupt the formation of HAAs [47].

## Conclusion

The objective of this study was to evaluate the profile and the contents of polar and apolar heterocyclic aromatic amines in braised and flamed chickens in Burkina Faso. The results obtained showed a great variability in the profile of HAAs in the samples. All the polar heterocyclic aromatic amines investigated were detected in the flamed chicken, while 4,8-DiMeIQx was not obtained in any of the braised chicken samples. As for the apolar HAAs investigated, they were all detected in braised and flamed chickens. The different concentrations obtained showed a high variability. Investigations on the content of precursors in raw chicken, the effect of marinating ingredients on the formation of HAAs should be conducted to elucidate this variability in the profile of HAAs and to control their occurrence.

## Supporting information

S1 Data (XLSX)
